# Multifunctional Smart Bone Implants: Fiction or Future?—A New Perspective

**DOI:** 10.3389/fbioe.2022.912081

**Published:** 2022-06-08

**Authors:** Inês Peres, Pedro Rolo, Marco P. Soares dos Santos

**Affiliations:** Department of Mechanical Engineering, Centre for Mechanical Technology and Automation (TEMA), University of Aveiro, Aveiro, Portugal

**Keywords:** smart implants, instrumented medical device, implant technology, bioelectonic implants, biointegration

## Abstract

Implantable medical devices have been developed to provide multifunctional ability to numerous bioapplications. In the scope of orthopaedics, four methodologies were already proposed to design implant technologies: non-instrumented passive implants, non-instrumented active implants, instrumented passive implants and instrumented active implants. Even though bone replacements are among the most performed surgeries worldwide, implant failure rates can still exceed 10%. Controversial positions multiply in the scientific community about the potential of each methodology to minimize the burden related to implant failures. In this perspective paper, we argue that the next technological revolution in the field of implantable bone devices will most likely emerge with instrumented active implants as multifunctional smart devices extracorporeally controlled by clinicians/surgeons. Moreover, we provide a new perspective about implant technology: the essence of instrumented implants is to enclose a hybrid architecture in which optimal implant performances require both smart instrumentation and smart coatings, although the implant controllability must be ensured by extracorporeal systems.

## 1 Background and Significance

The widespread of Implantable Medical Devices (IMDs) highlights their worldwide societal impact. In the scope of orthopaedics, technology innovation has triggered the development of four methodologies to design intracorporeal biomedical devices: 1) non-instrumented passive implants, only designed to restore mobility and reduce pain, and performance optimization based on geometry and materials; 2) non-instrumented active implants, focus on (bio)chemical modifications of surfaces; 3) instrumented passive implants, designed to monitor biomechanical quantities *in vivo*; 4) instrumented active implants, which are multifunctional smart implants incorporating therapeutic actuation system, bone-implant interface sensing system, processing system, wireless communication system and electric self-powering system. Although only non-instrumented passive and active technologies are available in the market, no agreement was achieved so far concerning the best methodology to minimize implant failures. At stake are millions of total hip replacement (THR) and total knee replacement (TKR) surgeries performed per year worldwide (Ferguson et al. (2018); Price et al. (2018)), as a result of an increasing trend registered both in developed and emerging countries over the last decades mainly due to osteoarthritis, a musculoskeletal disorder with a global disability burden around 4%. These incidences, and estimations stating they probably will double in the next decade, remain impressive (Ferguson et al. (2018); Price et al. (2018)). Although the THR has been recognized as “the operation of the [last] century,” implant failures exceeding 10% and according to demand patterns of about 6% after 5 years and 12% after 10 years following primary replacement have been reported. Indeed, important advances in bone implant technology have emerged throughout the last 20 years, but no significant reduction of revision rates have been reported (Ferguson et al. (2018); Price et al. (2018)). This is a major concern for scientists, entrepreneurs, clinicians and politicians. All are aware of the great impact of clinical outcomes of THR and TKR in health and social care systems, as well as the unfulfilled expectations of more than 60% of patients (Price et al. (2018)). The societal problem emerges more clearly when the current and future revision burdens, mainly in young and active patients, are considered. Around 30% of the overall patients are currently young (35% increase in the last decade), and sustained increases that can exceed 60% are expected in the next decade (Kurtz et al. (2009)). Besides, other relevant clinical outcomes claim for optimized longevity of these implantable devices: 1) the risk of revision surgeries is significantly higher with younger age groups (Price et al. (2018)); (b) surgical revisions are usually more complex and significantly invasive; (c) the probability to undergo a second revision is five to six-fold higher after the first revision; (d) the probability to develop a major postoperative complication is higher than 77% for patients with comorbidities; (e) 90-days mortality rates of young patients have already achieved 0.5%. And worse clinical outcomes have been issued for other replacements (shoulder, ankle, elbow, etc.). Therefore, a major challenge must be addressed: to develop revision-free implants. However, impacting breakthroughs towards the development of such implants require to focus the research efforts on the best technological methodology to design effective implant technologies with ability to fulfil high-demanding lifetime requirements (exceeding 2 decades (Ferguson et al. (2018); Price et al. (2018); Soares dos Santos et al. (2019))). Ultimately, the most fundamental requirement is to design implant technologies with ability to perform trajectories from failures states to non-failure states without disturbing the everyday life of patients (Ferguson et al. (2018); Soares dos Santos et al. (2015)).

Osseointegration is an essential process to establish an asymptomatic and stable long-term fixation (Sumner (2015)). An accurate control of the factors modulating the biointegration process is mandatory for performance optimization of implants, which must be accomplished at the micrometer and nanometer scale levels. As adverse bone remodelling intensifies, mainly due to wear debris and stress-shielding, the relative motion between the implant and bone increases, which can result in aseptic loosening (Sumner (2015)). Revision rates related to stress-shielding-induced bone loss can exceed 50%, incidences confirming the implant loosening among the most common causes indicated for THR (Ferguson et al. (2018); Price et al. (2018)). The periprosthetic infection is also a major consequence of implant insertion, currently rated as the most common indication for TKR and the third most common reason for THR. Current implant technology have shown clear evidences of their inability for revision-free replacements. The increasing societal and personal burdens associated to revision procedures, mainly performed in younger and/or active patients, highlight the importance of developing high-performance implant technology for long-term survival.

This paper then provides a deep discussion concerning the next technological revolution in the field of implantable bone devices. All analyses will be focused on uncemented implants, as: (i) long-term cemented fixations are harder to achieve; (ii) an increasing number of young and active patients is expected for the forthcoming decades; (iii) periprosthetic interfaces with bone-implant biocontact are easier to control. Our conclusions can be generalized for a wide range of implantable medical devices, including for orthopedics, neurology, ophthalmology and psychiatry.

## 2 Before Multifunctional Smart Implants

Current methodologies used to improve the performance of these bone implants have been based on the optimization of their geometry and materials (Sumner (2015)). Implant geometry plays a significant role in reducing the stress/strain-shielding, a mechanical phenomenon characterized by a lower mechanical stimuli delivered to the peri-implant tissues following implant insertion, which can render adverse bone remodeling and even implant failure by aseptic loosening (Sumner (2015)). Recent breakthroughs already allow to design implants with custom-made geometries and nanometer-scale textured surfaces to improve primary stability, enhance secondary stability and improve bone-implant fixation. The mismatch between mechanical properties of bone and bulk materials is also critical to improve stress/strain distributions (Sumner (2015)). Several methods have been proposed to minimize these differences, among which the use of composite materials, porous materials and multi-material structures must be highlighted.

Chemical and biochemical modifications of the implants’ surfaces have been considered the most effective methodology to design non-instrumented active implants (Devgan and Sidhu (2019)). Two generations of coating materials have already emerged. The first one was based on bioactive materials, including bioceramics, biometals and biopolymers to enhance bone-implant bioactivity and bonding. The second generation has been focused on biomaterials to promote specific biointegration cellular responses for therapeutic actuation along the bone-implant interface. A wide range of coatings have been researched to enhance osseointegration, while simultaneously ensuring non-cytotoxicity and non-genotoxicity, such as calcium phosphate-like coatings, Carbon/carbon fiber reinforced coatings, bioactive glass coatings, bio-mimetic coatings, nanostructured coatings, anti-infection coatings, biomolecule coatings, drug-loaded coatings (e.g., for anti-bacterial agents delivery, growth factor delivery, anti-inflammatory and immunosuppressing drug delivery, gene therapy and nucleic acid delivery, antiresorptive drug delivery, anticancer drug delivery) and multifunctional coatings (both to enhance osseointegration and to prevent infection).

The concept of Instrumented Implant is another approach that aims to optimize the performance of implants (Soares dos Santos et al. (2019; 2015)). Besides their inherent function in replacing bone and performing load bearing functions, the main idea has been to engineer new types of implants incorporating inner electronics and instrumentation to perform sensing and therapeutic actuations along the bone-implant interface. Although it is an unquestionably disruptive concept, very few instrumented passive systems were implanted in humans. By designing them embedding wireless communication, monitoring and non-autonomous powering systems, several biomechanical quantities (forces, moments, deformations and temperatures, etc., along the implant) were already measured *in vivo* (Soares dos Santos et al. (2019)). The development of hip, knee, shoulder and spine instrumented implant are some examples of successfully research projects carried out for specific goals: 1) *in vivo* data collection to optimize biomechanical models; 2) optimization the implant designs and materials; 3) performing preclinical testing of new implant technologies; 4) monitoring the rehabilitation process after implant insertion.

Although all these technological breakthroughs hold potential for future implementation of high sophisticated biodevices, the most effective methodology to ensure revision-free outcomes is not obvious to identify. Indeed, clinical outcomes have not been able to suggest the ineffectiveness of passive implants or the effectiveness of active implants. Besides, no strong evidences explain why research must be focused on geometry and biomaterials rather than in the design of instrumented implants. In order to emphasize what research lines must be primarily pursued to implement failure-free bone implants, the potential of each methodology to design implants capable of optimal performances was recently inferred. By conducting an optimality analysis to passive and active implants using the Pontryagin Maximum Principle, it was demonstrated that optimal implant performances require some kind of sensing, actuation, communication and self-powering systems (Soares dos Santos et al. (2015)): to change a state of implant failure to non-failure states according to optimal trajectories, a biological and/or non-biological feedback control loop is mandatory. This finding predicts that pre-optimization of geometries and surfaces texture is not enough: implants must provide some active operations over the bone-implant interface. Concerning non-instrumented active implants, therapeutic actuations using bioactivity promoted by current biomaterials present significant limitations, namely:

i) Their design can be very complex, increasing as their multifunctional ability increases.
ii) Controllability of the bone-implant interface behaviour is rather reduced: (a) the delivery dynamics does not consider the bone-implant (biochemical and biomechanical) states; (ii) bioactivity, osteoconductivity and osteoinductivity cannot be changed after implant insertion (pre-established actuation mode); (c) long-term release of bioactive substances (drugs, biomolecules, etc.) most likely will be quite hard to implement and according to non-personalized patterns.
iii) The ability to deliver different stimulations to target tissue peri-implant regions will most likely be quite difficult to attain.
iv) Time-dependent dosing release can be significantly hard to monitor.

Current non-instrumented active implants are designed without implant-clinician communication. Monitoring of the bone-implant fixation cannot be performed throughout the daily life of patients as only imaging methods (radiography, arthrography, scintigraphy, etc.) are used (Cachão et al. (2020)). Even though this imaging-based monitoring is able to detect failure and non-failure states, feedback control of the biointerface state is restricted to non-real-time drug administration. Even so, this methodology allows the design of autonomous implants comprising biological sensing, actuation, communication and powering, operating together in closed-loop feedback. Nevertheless, to our knowledge, no research has been carried out in this scope, most likely due to the high complexity involved. Another very recent approach aims to develop non-instrumented biomaterial-based communication system, sensors and actuators. An electronic circuit for intrinsic communication between sensors and actuators was printed on Ti6Al4V substrate using smart additive technologies (Moura et al. (2020)). Although this is a very promising approach, fundamental science still has a long way to go before the development of silicon-free multifunctional processing units and conditioning circuitry for sensing and actuating systems.

**FIGURE 1:**
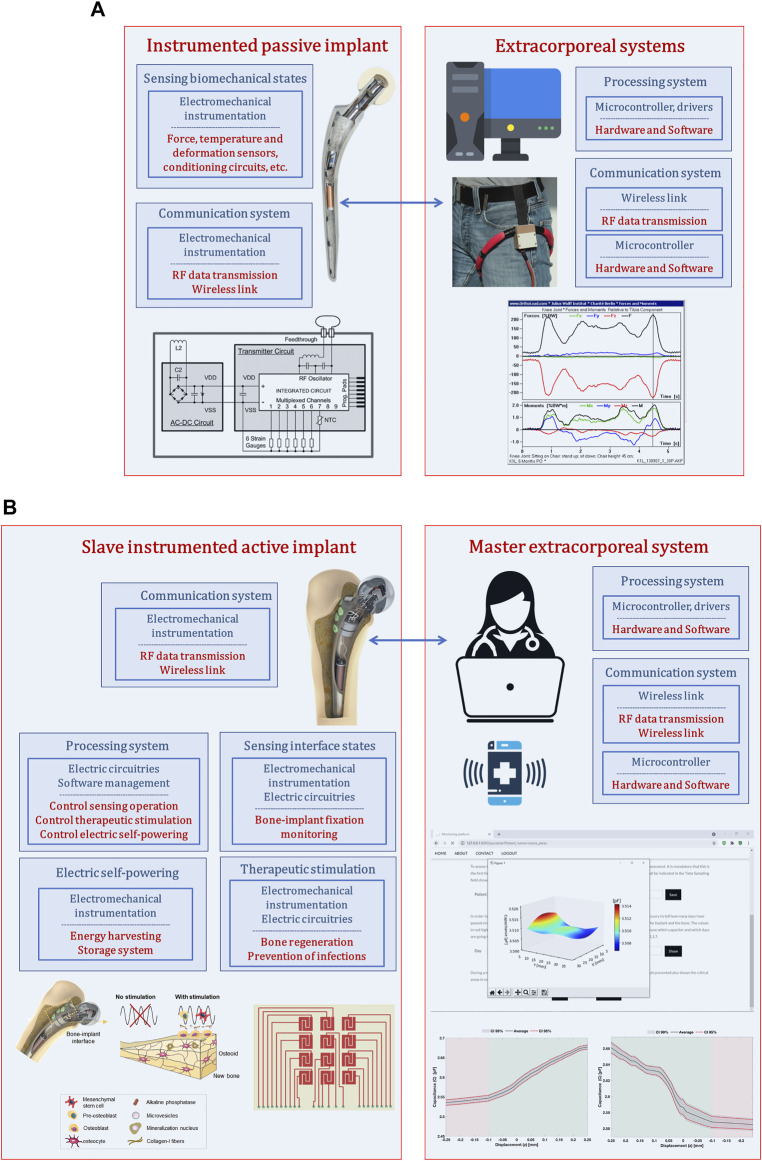
**(A)** Architecture used to design instrumented passive implants (Soares dos Santos et al. (2015; 2014)); **(B)** Architecture used to design instrumented active implants as multifunctional smart devices (Soares dos Santos et al. (2019; 2015); de Sousa et al. (2021)).

Although instrumented passive implants strongly contributed to the development of smart implants, they did not bring about a technological revolution in the field of implantable bone devices. Their monitoring systems were not designed to support therapeutic actuations in peri-implant regions (Figure 1A): their focus on measuring biomechanical quantities established a very restricted operation border. Certainly that these instrumented implants can also include smart coatings, but force, temperature and deformation transducers were not embedded into the implants to enhance a smarter bioactivity. A real implant-clinician communication is not intended, just data transfer between electronic circuitries inside and outside human body mainly for non-therapeutic purposes (albeit data acquisition can be used to provide non-real-time drug administration throughout rehabilitation after surgery). Moreover, their non-autonomous electric powering make bone-implant fixation states impossible to follow up in a personalized basis during daily life of patients. Finally, as they were not designed incorporating instrumentation to perform therapeutic trajectories from loosening to fixation states (Soares dos Santos et al. (2014)), no closed-loop software-based feedback can be carry out, and, hence, they can only aspire to provide similar performances to those ensured by non-instrumented active implants.

## 3 Emerging of Multifunctional Smart Implants

Instrumented active technologies has emerged as a leading research topic that aims to design implants comprising biophysical therapeutic actuation, bone-implant interface sensing, implant-clinician communication and self-powering ability (Figure 1B) (Soares dos Santos et al. (2021; 2019; 2015)). Research teams working on these new smart multifunctional implants aims to design them incorporating electromechanical instrumentation, electrical circuitry and software management to allow personalized therapeutic actuations along the bone-implant interface controlled by clinicians/surgeons throughout everyday life of patients (without troubling their quotidian activities) (Soares dos Santos et al. (2021; 2019; 2015)). This approach establishes a Master-Slave distributed architecture, in which the extracorporeal system is designed as a Master system with ability to control the operation of the multifunctional implant, now designed as a Slave technnology (Figure 1B). Therefore, clinicians/surgeons will be able to define: (i) personalized therapies to deliver in the bone-implant interface, such as waveform, magnitude and frequency of stimuli, daily stimulation exposure, resting time and total therapy duration (Soares dos Santos et al. (2019; 2016)); (ii) personalized sensing of the bone-implant interface, namely sensing periodicity and target peri-implant regions to be monitored (Soares dos Santos et al. (2021)). The capability of osteogenic, osteoconductive and osteoinductive optimized stimulations are thus inherent to instrumented smart implants. The ultimate goal is to personalize therapies to patients of all ages and according to their idiosyncrasies, such that revision surgeries can be effectively minimized.

As a superior controllability of osseointegration will be obtained if bone-implant interface states are monitored (Soares dos Santos et al. (2015)), a proliferation of studies in this scope has been published. Most of the proposed technologies focused on five methodologies: vibrometric, acoustic, bioelectric impedance, magnetic induction, and strain (Cachão et al. (2020)). Although none has already been implanted in human patients, most of them: (a) are able to operate non-invasively and with minimum interaction with peri-implant tissues; (b) can be designed for different geometries of implant surfaces; and (c) can follow-up the bone-implant interface state throughout the daily life of patients. Even more impacting has been the proposal of a cosurface capacitive technology with ability to detect a wide range of bone-implant fixations, from strong bonding to severe loosening (Soares dos Santos et al. (2021)). Moreover, in addition to the advances engineered by previous technologies, cosurface capacitive sensing can provide a much higher controllability and personalized monitoring of peri-implant target regions/tissues. Indeed, higher sensibility is reported to detect small-scale debonding disorders, which is an important feature to identify early loosening states; nevertheless, different loosening states can also be detected (Soares dos Santos et al. (2021)). Moreover, sensing can be provided by customized capacitive networks such that detection, controlled by extracorporeal informatic systems, can be extended over very small-scale implant surface areas up to the entire surface (Peres et al. (2022)). Two other important advantages are supported by these capacitive sensing systems. One concerns their electric powering: very low electric currents and voltages lower than 10 V are required, hence opening true opportunities for replacing conventional power systems (batteries) by energy harvesting technologies, mainly by those converting body biomechanical motion into electric energy (Vidal et al. (2021); Soares dos Santos et al. (2016a), Soares dos Santos et al. (2016b); Carneiro et al. (2021)). The other concerns the ability of capacitive network sensors to effectively perform as a hybrid sensing-acting system. Feasibility of capacitive architectures overcome sensing operation. Similar capacitive designs have also been used as therapeutic stimulation systems, revealing promising performances based on the delivery of controllable/personalized electric field stimuli (waveform, strength, frequency, periodicity, daily stimulation exposure, etc.), and according to target-oriented cytotoxic- and genotoxic-free stimulation of peri-implant regions (Soares dos Santos et al. (2019); de Sousa et al. (2021)). Already achieved results have been highlighting their effectiveness promoting osteoconduction and osteoinduction (de Sousa et al. (2021)), which makes them very attractive to obtain optimized trajectories of bone matrix formation and maturation and bone matrix mineralization. Different non-capacitive electrode-based stimulators have also been employed to deliver electric fields to implant-bone interfaces experiencing bone necrosis, such as stimulation electrodes attached to the implant surface and connected via a coil inside an insulation layer (Zimmermann et al. (2021)), as well as to deliver electric currents to infected implant-bone interfaces with *Staphylococcus aureus* (the main pathogen for infections associated with metallic implants), such as the three-electrode cathodic voltage-controlled electrical stimulation (Ehrensberger et al. (2015)).

Self-powering technologies are mandatory to electrically supply all instrumentation and electronics inside implantable bioelectronic devices. The ability of energy harvesters to supply sufficient and continuous energy remains a critical problem in the scope of implantable medical devices. An even superior performance is demanded for intracorporeal multifunctional ability: everlasting capability to simultaneously power intensive monitoring, processing, actuation and communication operations. Research has been mainly conducted towards the design of innovative transduction mechanisms based on electromagnetic, piezoelectric and triboelectric principles to convert mechanical energy into electric energy. The low energy efficiencies for low-frequency mechanical excitations (typical in body motions) remain an unsolved problem. Besides, small-scale electromagnetic harvesters are not currently able to harvest voltages exceeding 10 V for low-frequency-excited operation. Piezoelectric energy harvesting are being investigated to overcome this limitation by incorporating stacked piezoelectric elements with multiple layers (Lange et al. (2021)), even though such harvesting technologies (as well as triboelectric harvesters) behave as low current sources, jeopardizing the processing capability to run intensive management and control algorithms. The design of hybrid electromagnetic-triboelectric and electromagnetic-piezoelectric harvesters are novel approaches emerging to simultaneously take advantage of their complementarity, i.e., harvest high voltages (provided by triboelectric or piezoelectric generators) and high electric currents (provided by electromagnetic generators) (Vidal et al. (2021)). It is true that none of these hybrid generators were validated so far to power smart implants, but they hold potential for the implementation of a new generation of small-scale smart energy generators for personalized self-powering.

**FIGURE 2:**
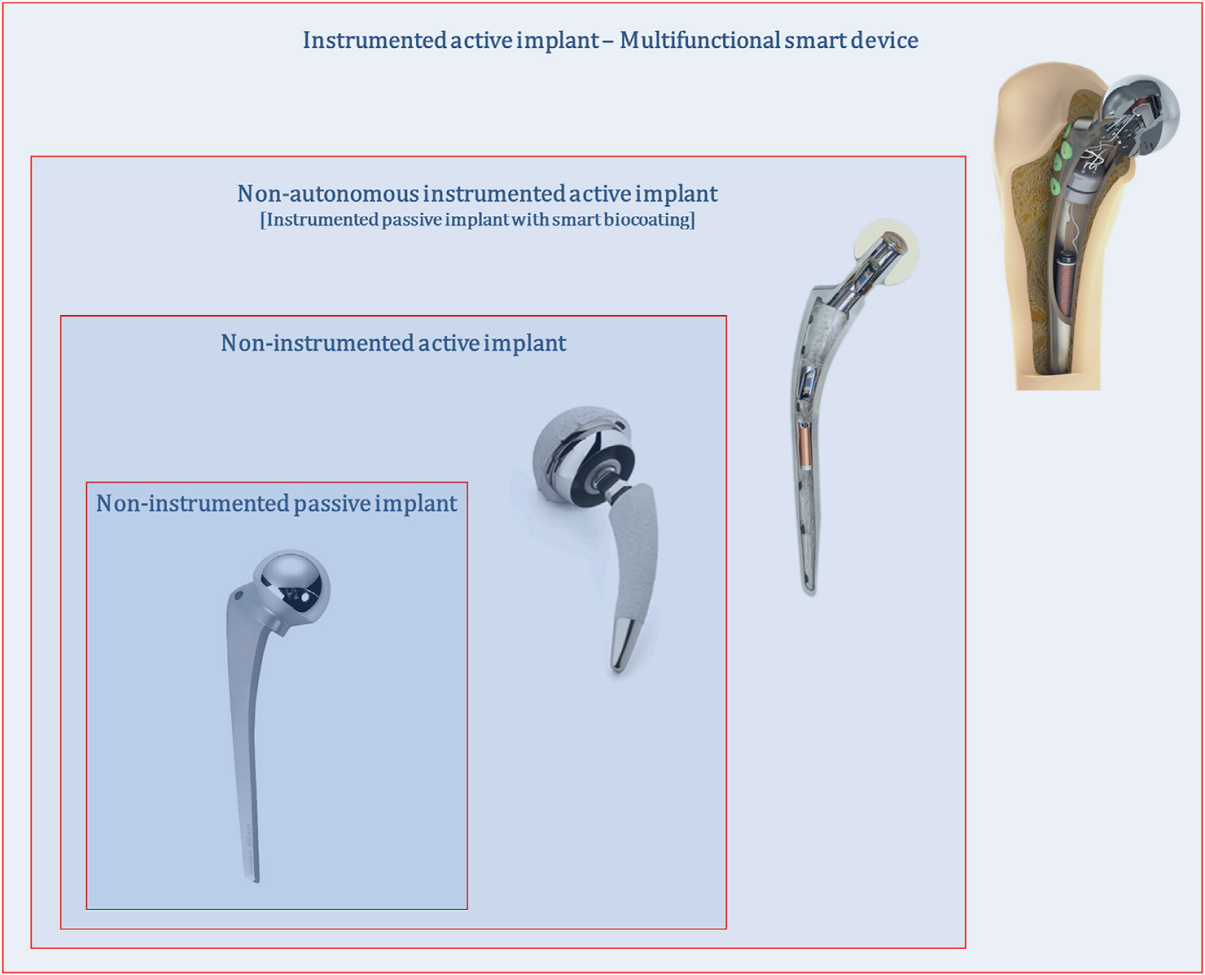
Multifunctional smart implants as hybrid technologies framing non-instrumented passive, non-instrumented active and instrumented passive implant technologies.

Up to today, no miniaturized processing system was already developed to manage sensing, stimulation and self-powering operations, most likely because fundamental research is still being conducted to explore the effectiveness, feasibility and controllability of sensing and stimulation systems. It should be noted that all instrumentation and electronics incorporated within smart implants can be disabled, such that they only operate as non-instrumented passive or non-instrumented active implants. In fact, instrumented implants will only perform optimal trajectories from failure states to non-failure states if implant geometries, materials and textures are optimized. Moreover, multifunctional instrumented active implants are not technologies apart from non-instrumented active implants: the former includes the latter (Figure 2). The true essence of instrumented implants is to enclose a hybrid architecture in which optimal implant performances require both smart instrumentation and smart coatings, although the implant controllability is ensured by the Slave-based instrumentation and, hence, by clinicians/surgeons extracorporeally controlling it using the Master system. Hence, failure-free bone implants demand research lines primarily pursuing such smart hybrid technologies: multifunctional instrumented implants require all previous implant concepts; all previous implant technologies fulfil their goals being framed in multifunctional instrumented implants.

## 4 Final Remarks

Multifunctional smart implants establish a wide range of new means of therapeutic actuations. The expertise of clinicians/surgeons can be used to administrate appropriated stimulatory therapies, taking advantage of decision-making based on previous research studies and on outcomes obtained from other patients. Further sophistication may be attained if active implants are designed with the ability to autonomously define the stimulatory therapies. Nowadays, available technology allows transferring the human knowledge to data storage systems located inside instrumented implants, and artificial intelligence algorithms can learn from the inherent variability of bone-implant interface states of each patient. Smart therapeutic actuation can be explored as a time-dependent complementarity: biophysical stimuli delivery can be exploited as the main method to avoid implant failures, although it can be programmed as an adjuvant strategy to enhance the therapeutic ability of smart biocoatings. The capability for osteogenic, osteoconductive and osteoinductive stimulations are thus inherent to instrumented active implants.

This new concept model of Instrumented Active Implant as a hybrid technology also presents some constraints. The decision-making of clinicians/surgeons is not error-free, and the probability to be administrated non-optimized therapies increases as the number of monitored bone-implant states decreases. Other risks cannot be disregarded, such as the cytotoxic and genotoxic risks related to implant fractures, which are significantly higher when implant systems are biointegrated in young/active patients. Biocompatible cosurface stimulators can be already constructed, but manufacturing of biocompatible sensing, processing, energy storage and communication systems is still a hot topic in chemistry and material sciences. Risks can be minimized if instrumentation and electronic systems were fully encapsulated using biocompatible materials, as well as by miniaturizing these inner systems to minimize the hollowed structures inside implants. The higher the number of sensing/acting structures, the more complex electronic systems will be. Therefore, the most reliable scheme seems to be the incorporation of sensors and stimulators in the peri-implant regions where more pronounced bone loss usually occurs (such as the proximomedial region of total hip replacements). Another underestimate issue is the inherent osteogenic effects that can be promoted by extracorporeally-induced magnetic fields stimulating the bone-implant interface when communication and/or powering operations are performed. Although it seems reasonable to assume much lower exposure times to these stimuli when comparing with those delivered for therapeutic actuation, these extracorporeally-originated stimuli can interfere with administrated control trajectories imposed to bony proliferation, differentiation and mineralization. Finally, although smart implants will be more expensive than passive implants, the societal burden will be significantly reduced, as the number of years lived with disability will most likely be minimized.

Several challenges will arise in the development of instrumented active implants. Promising results were achieved concerning the performance of cosurface capacitive stimulation to control bone remodeling, but the osteogenic effects of a wide variety of stimuli are still unknown. Idiosyncrasies of patients may difficult the identification process towards optimality of therapeutic actuations. The therapeutic complementarity approach between biophysical stimulation and active biocoating stimulation will require deep multidisciplinary research, in particular in patients suffering from severe bone remodeling disorders (e.g. osteoporosis). To ensure everlasting operation of instrumented implants, smart adaptive self-powering systems must be designed, since the performance of electromagnetic harvesters is function of time-varying body motion dynamics driving the harvesters. Moreover, such harvesters must be designed considering geometric optimization prior to fabrication, taken into account the tridimensional body motions during the routine activities of patients and according to their ageing processes. Indeed, the amount of energy harvested may impose limits to the magnitude of biophysical stimuli that can be delivered to the peri-implant bone volume and, consequently, the amount of therapeutic actuation trajectories may be reduced. Storage systems will most likely be necessary, mainly to power implants during the perioperative period, in old ages and when demanding therapies are required (e.g., several hours of daily stimulation). Finally, as these implants always have to be manufactured with inner hollowed structures, it is imperative to research new materials with bulk properties that can minimize fracture risks. An overview of the main strengths and limitations of multifunctional smart implants is presented in Table 1, which also includes the main challenges that must be overcome to ensure their effective development and clinical translation.

**TABLE 1:** Overview of the main strengths and limitations of multifunctional smart implants as hybrid technologies, as well as the main challenges to their effective development.

Analyses	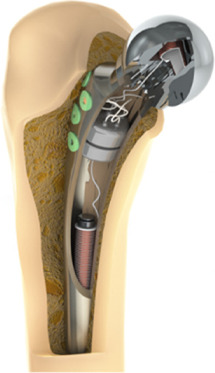
Strengths	Revision-free implants: ability to provide superior performances ensuring long-term survival.
	Customized performance: (i) ability to provide personalized therapeutic actuations throughout long time periods without disturbing the everyday life of patients; (ii) ability to enable or disable all instrumentation inside implants
	High controllability: a wide range (waveform, magnitude, frequency, periodicity, daily stimulation exposure, etc.) of time-dependent biophysical stimuli can be delivered to target peri-implant regions considering the bone-implant interface state
	Decision-making: performed by clinicians/surgeons or Artificial Intelligence Algorithms
	Therapeutic/sensing technology: (i) the same technologies can be applied both for therapeutic and sensing operations; (ii) ability to be customized for different implant types and designs; (iii) ability to provide therapies for several bone-implant interface conditions, including both septic and aseptic loosening
	Therapeutic complementarity: delivery of biophysical stimuli can be programmed either as the main therapeutic method or adjuvant
Limitations	Therapeutic error: decision-making performed both by clinicians/surgeons or Artificial Intelligent algorithms are not error-free
	Risks related to incorporated instrumentation: cytotoxic and genotoxic risks can occur due to implant fractures
	Optimal performance requirement: optimal trajectories from failure states to non-failure states require: (i) optimized implant geometries and materials, including smart coatings; (ii) optimized communication systems; (iii) smart self-powering systems.
	Instrumentation complexity: the higher performance requirements, the more complex instrumentation and electronic systems will be
	Electric power requirements: the higher number of (therapeutic, sensing and processing) operations are required, the more complex the self-powering system will be
Challenges	How to find optimal performances: (i) identification of the optimal biophysical stimuli considering idiosyncrasies of patients; (ii) design smart biocoatings
	How to provide therapeutic complementarity: interfunctional coordination between smart biophysical stimulation and smart biocoating stimulation.
	How to engineer effective implants’ architectures: (i) design of hollowed structures minimizing fracture risks; (ii) miniaturization and encapsulation of all instrumentation inside implants
	How to engineer electric power generation: design of smart adaptive self-powering systems considering time-varying body motion dynamics
	How to ensure autonomous operation: design of Artificial Intelligence algorithms for therapeutic decision-making

Despite constraints and challenges to be addressed in future research, there are strong indicators highlighting a coming technological revolution in the field of implantable bone devices: implant technologies emerging as hybrid instrumented multifunctional devices with ability to promote both smart biophysical stimulation and smart active biocoating stimulation.

## Data Availability

The raw data supporting the conclusion of this article will be made available by the authors, without undue reservation.
